# The Effect of Optical Flow Motion Direction on Vection Strength

**DOI:** 10.1177/2041669519899108

**Published:** 2020-01-13

**Authors:** Yoshitaka Fujii, Takeharu Seno

**Affiliations:** Faculty of Design, Kyushu University, Fukuoka, Japan; Research Organization of Open Innovation and Collaboration, Ritsumeikan University, Osaka, Japan; Faculty of Humanities and Social Sciences, Kumamoto University, Kumamoto, Japan; Faculty of Design, Kyushu University, Fukuoka, Japan

**Keywords:** vection, direction effect, oblique effect, asymmetry, motion perception

## Abstract

In some phenomena of visual perception, the motion direction of visual stimuli can affect perception. In particular, asymmetries between oblique directions and cardinal (horizontal and vertical) directions have been reported and are known as *oblique effects* (e.g., contrast sensitivity and motion threshold). In this study, we investigated how vection strength depends on motion direction. Participants observed random-dot optical flow in a circular field and rated the perceived vection strength. Dot movement was systematically controlled using the following angles: 0° (up), 30°, 45°, 60°, 90°, 120°, 135°, 150°, and 180° (down). We found that vection strength depended on motion direction and was weaker in the oblique directions than cardinal directions. Thus, the effect of motion direction on vection strength was variable, as seen in the shape of the oblique effect.

## Introduction

Stationary observers perceive self-motion when a moving visual stimulus is presented in their visual fields. This illusory self-motion is termed *vection*, and the visual stimuli are termed *optical flow*. Numerous characteristics of vection have been reported since Brandt, Dichgans, and Koenig first investigated this interesting illusion in 1973.

On the earth, all motion is affected by gravity and most motions occur on the ground. Therefore, vertical and horizontal directions (termed *cardinal directions* along with forward and backward) are very important for controlling the body, such as when standing vertically or walking on a horizontal floor. Especially for vection, the direction of gravity is an important factor. Several studies have shown that vection is closely related to and depends on the posture of the observer in relation to the direction of gravity, such as sitting, lying down (Kano, 1991), tilting (Nakamura & Shimojo, 1998), upside down, or right side up (Mori & Seno, 2017). In this study, we investigated the effect of motion direction on vection.

The close relationship between vection and gravity can produce asymmetrical effects of motion direction on vection strength because gravity always pulls in one direction. Some studies have focused on asymmetry of vection in the frontal parallel plane, reporting that vertical motion induces stronger vection than horizontal motion (Ito, 2004; Ito & Fujimoto, 2003; Kano, 1991; Trutoiu & Mohler, 2009). Keshavarz, Speck, Haycock, and Berti (2017) also compared vection strength between horizontal and vertical motion directions using four display systems (a dome-shaped projection screen, a flat projection screen, a combination of three computer displays, and a single computer display). They showed that horizontal motion induced stronger vection than vertical motion only when using the domed screen. They suggested that the effect was caused by the curved shape of the screen and concluded that the effect of motion direction was not a crucial factor in vection perception. Studies have found conflicting findings related to asymmetry in vertical directions (up vs. down). Kano (1991) reported that downward flow induced stronger vection than upward flow, but Trutoiu and Mohler (2009) and Seya, Shinoda, and Nakaura (2015) reported the opposite.

Other studies have compared direction effects between forward and backward motion, indicating that backward self-motion (contracting optical flow motion) induced stronger vection than forward self-motion (expanding optical flow motion; e.g., Andersen, 1986; Bubka, Bonato, & Palmisano, 2008; Ito & Shibata, 2005; Reinhardt-Rutland, 1982; Seno, Ito, Sunaga, & Nakamura, 2010).

In the real world, in addition to vertical and horizontal planes (cardinal directions), objects also move in oblique directions. The visual system in the brain sometimes performs differently in oblique and cardinal directions, and this asymmetry is known as the *oblique effect* (for a review, see Appelle, 1972). Oblique effects have been found in a wide variety of visual perceptions, including spatial resolution, contrast sensitivity, detection, discrimination, recognition, and memorization (Appelle, 1972; Campbell, Kulikowski, & Levinson, 1966; Essock, 1980; Howard, 1982). Typically, the performance in cardinal directions is better than in oblique directions. This is thought to be because the visual system distributes more resources to cardinal directions because they are more important (e.g., gravity is vertical and flat ground is horizontal).

Many studies have shown that oblique effects occur when the head or body is tilted. Examples can be found in visual search (DeFord, Prather, & Essock, 1998, 1999), orientation–discrimination (Attneave & Olson, 1967; Buchanan-Smith & Heeley, 1993; Chen & Levi, 1996; Ferrante, Gerbino, & Rock, 1995; Orban, Vandenbussche, & Vogels, 1984), contrast sensitivity (Banks & Stolarz, 1975; Lennie, 1974), and vernier acuity (Corwin, Moskowitz-Cook, & Green, 1977; Saarinen & Levi, 1995).

These studies indicate that in addition to vection, oblique effects also depend on the direction of gravity and imply that vection strength has an oblique effect. Although to our knowledge, none have compared vection strength between oblique and cardinal directions. However, some studies have focused on the asymmetry of oblique directions in vection. To investigate the effect of visual field, Telford and Frost (1993) measured vection strength not only in the vertical and horizontal directions but also in the oblique directions. They found that vertical flow induced the strongest vection and that differences between horizontal and oblique directions were not clear. However, their stimuli were not symmetrical (optical flow stimuli were drawn in rectangle field with longer widths), and this could have caused asymmetrical vection strength. In addition, they used their own custom indices of vection strength that are not common; participants synchronized a computer mouse to the perceived self-motion, and the mouse speed and latency were used as vection indices. In another study, Ito and Fujimoto (2003) used an integrated flow of vertical and horizontal motion and, based on their results, conjectured that vection strength in the oblique direction was stronger. However, the effect of oblique directions was not comprehensively discussed in either of these studies because vection strength when stimuli move in oblique directions was not the main topic in either case.

Vection is closely related to perception of visual motion. Global motion of optical flow which induces vection is initially processed in low-level motion detector, which is common between vection and visual motion perception. Past studies have argued that some types of motion perception imply the existence of oblique effects. For example, Gros, Blake, and Hiris (1998) and Dakin, Mareschal, and Bex (2005) showed that the threshold for detection and direction discrimination of coherent motion indicates oblique effects. Matthews and Qian (1999) confirmed an oblique effect of direction discrimination threshold and showed that speed discrimination threshold was not associated with any oblique effect. Thus, asymmetry of motion signals can induce asymmetrical vection.

In this study, our primary goal was to investigate the effect of motion direction on vection. Vection strength was measured in various directions, including oblique and cardinal directions. To determine whether the direction effect can be modified by oblique motion, vection strength was compared between cardinal and oblique directions (Experiment 1). We also investigated whether the effect of motion direction on vection is caused by the intensity of the motion signal (Experiment 2). In both experiments, visual stimuli were presented in a circular field and were symmetric in all directions. This allowed us to directly compare motion-direction conditions without any effects related to the asymmetry of stimuli. In addition, we employed commonly used indices of vection strength, which differed from the unconventional measures found in Telford and Frost (1993). The indices we chose have been repeatedly used in the literature, making them useful for directly comparing our results to those from previous studies (see Procedure in “Experimental Method” subsection).

## Experiment 1

### Experimental Method

#### Apparatus

Stimuli were generated and controlled with Matlab (R2014b, Mathworks, USA) and Psychotoolbox-3 (Brainard, 1997; Kleiner, Brainard, & Pelli, 2007; Pelli, 1997) on a personal computer (ALIENWARE 17R1, Dell, USA), and presented on a plasma display (3D VIERA [TH-65AX800], Panasonic, Japan, 65-in. [165-cm] screen with a 1,920 × 1,080 pixel resolution and a 60-Hz refresh rate). Viewing distance was 57 cm. The experiment was conducted in a dark room and a chin rest was used to immobilize the heads of the participants.

Each pixel of the plasma display was a rectangle and arranged on a square-grid pattern as is usual on most displays. This means that the stimuli that were intended to be symmetrical were slightly asymmetric. However, the pixels were very small and stimuli were drawn by antialiased subpixels. Furthermore, the experimental stimuli induced suprathreshold motion signals. Therefore, any pixel asymmetry had very weak or no effect.

#### Stimuli

**Figure 1.**
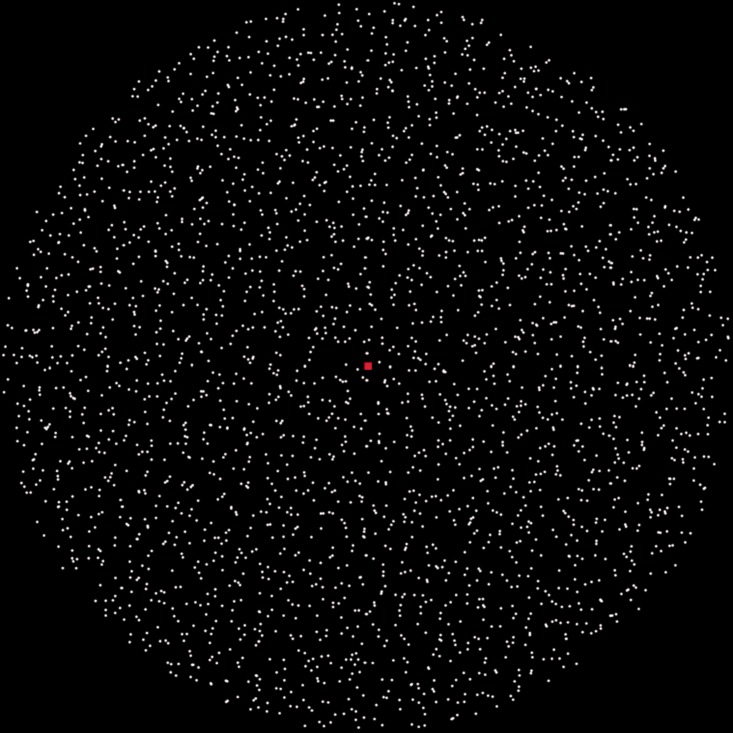
The stimulus used in Experiment 1 and Experiment 2.

Stimuli were random-dot optical flows (Figure 1; movies can be downloaded from http://www.senotake.jp/stimulus/2016/). White dots were placed in a circular field at the center of a black background. Dot luminance was 16.2 cd/m^2^ and that for the background was less than 0.01 cd/m^2^. Dot density was 0.54/deg^2^. The diameter of the central dot field was 82° and the size of each dot was 0.30°. Dots were antialiased. In addition, red fixation point was placed at the center.

All dots moved coherently in a single direction at a constant speed (33 deg/s), with a duration of 40 seconds. The angle of motion direction was varied over 16 conditions; 0° (up), ±30°, ±45°, ±60°, ±90°, ±120°, ±135°, ±150°, and 180° (down). The positive values indicate rightward motion, and negative values indicate leftward motion.

#### Procedure

Participants sat on a chair in a dark room and observed the stimuli on the display. They were instructed to focus on the fixation point throughout the viewing and to press and hold a button as soon as they perceived vection. This allowed us to measure vection latency and duration. After each stimulus presentation, subjective vection strength was measured via magnitude estimation. Participants rated vection strength using a 101-point scale that ranged from 0 (*no vection*) to 100 (*very strong vection*). Very strong vection meant that observers perceived vection as if they were actually moving in the scene very naturally by real locomotion. Thus, if participants perceived that they were moving about half as much as natural self-motion, they would rate vection strength as “50.” They were allowed to choose a rating greater than 100 if necessary, but none of them did so. This method is the same as we have used in several studies (Fujii, Seno, & Allison, 2018; Seno et al., 2010, 2017, 2018; Seno, Palmisano, Riecke, & Nakamura, 2015) and has been confirmed to be valid for evaluating vection strength.

Each participant completed four experimental sessions, with each session containing 18 trials: In each session, 0° and 180° conditions were presented twice and the other 14 directions were each presented once. This was done to balance the trial numbers among directions when bilaterally symmetrical conditions were identified. Within each session, trial order was random.

#### Participants

Thirteen adult volunteers participated in this experiment (students, faculty, and staff at Kyushu University, including the authors^1^; mean age = 27.9 ± 6.3 years, range of 22–38 years, 3 females and 10 males). All participants were healthy, had a normal or corrected-to-normal vision, and no history of vestibular system diseases. No one (aside from the authors) was aware of the purpose of the experiment.

#### Ethics statements

The experiment was preapproved by the Ethics Committee of Kyushu University and was conducted following the guidelines given in the Helsinki Declaration. Written informed consent was obtained from each participant before the experiment.

#### Statistical analysis

One-way analyses of variance were used to test whether motion direction significantly affected each separate component of vection strength (subjective magnitude, onset latency, and total duration). In addition, post hoc multiple comparison analyses (Ryan’s method) were performed to determine whether any differences between pairs of direction conditions were significant. To further investigate the oblique effect of vection strength, *t* tests were performed between three vection indices obtained during cardinal and oblique conditions.

### Results

**Figure 2.**
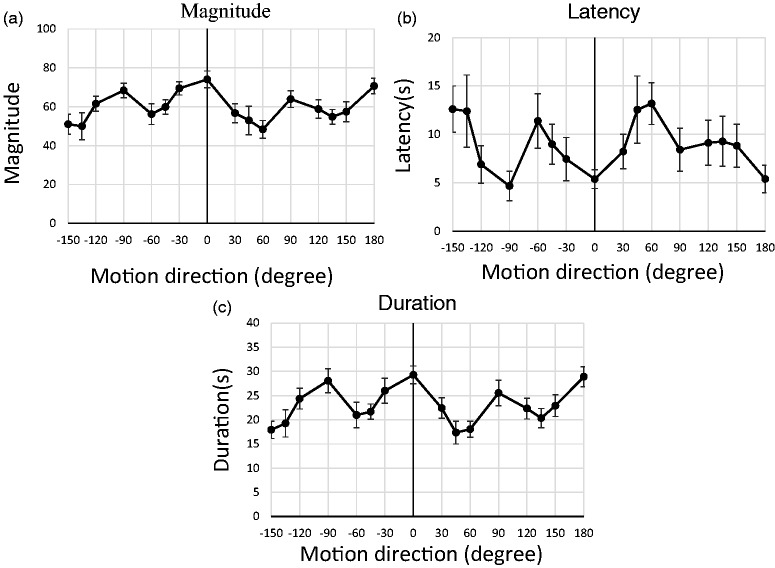
Results from Experiment 1. Vection strength was assessed via three indices: (a) subjective magnitude, (b) vection onset latency, and (c) vection duration. Error bars indicate standard error (*N* = 13).

**Figure 3.**
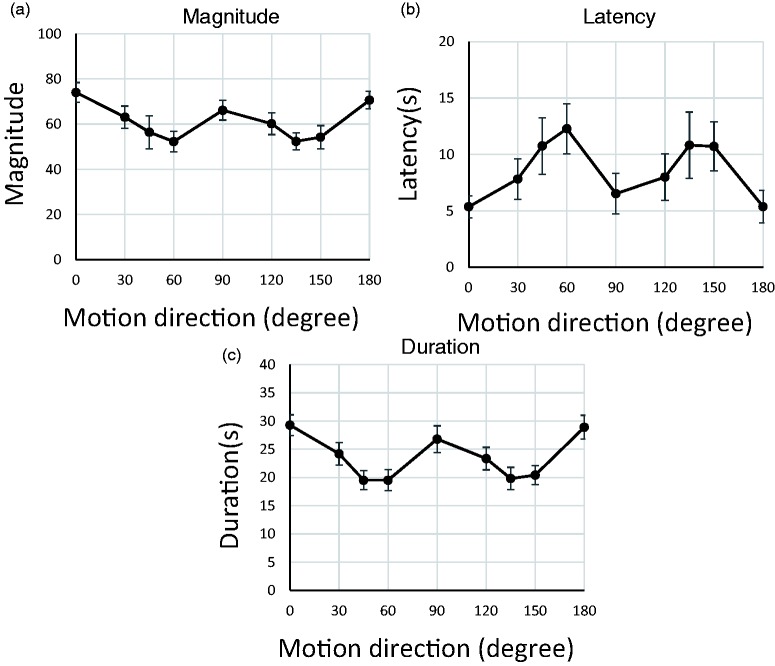
Results from Experiment 1 after averaging left/right symmetrical conditions. Vection strength was assessed via three indices: (a) subjective magnitude, (b) vection onset latency, and (c) vection duration. Error bars indicate standard error (*N* = 13).

The results (Figure 2) were almost the same for conditions with left/right symmetry (±30°, ±45°, ±60°, ±90°, ±120°, ±135°, and ±150°). Considering the bilateral symmetry of the visual system, and the fact that our main interest was the effect of motion-direction angle from vertical (not differences between left and right processing), we averaged the results from left/right symmetrical conditions (Figure 3) so that motion-direction effects relative to straight up could be more directly identified.

Figure 3 shows that motion direction affects vection strength. One-way analyses of variance for each measure revealed significant effects of motion direction for all three vection indices: magnitude: *F*(8, 96) = 6.63, *p* < .001, η^2^ = .21; latency: *F*(8, 96) = 5.18, *p* < .001, η^2^ = .10; and duration: *F*(8, 96) = 10.48, *p* < .001, η^2^ = .24. In addition, Ryan’s test for multiple comparisons indicated significant differences (*p* < .05) for magnitude (0° vs. 45°, 60°, 120°, 135°, and 150° conditions; 180° vs. 45°, 60°, 135°, and 150°), latency (0° vs. 45°, 60°, 135°, and 150°; 180° vs. 45°, 60°, 135°, and 150°; 90° vs. 60°), and duration (0° vs. 45°, 60°, 120°, 135°, and 150°; 180° vs. 45°, 60°, 120°, 135°, and 150°; 90° vs. 45°, 60°, 135°, and 150°). The other comparisons were not statistically significant.

The initial analyses indicate that vection strength depends on motion direction and the post hoc analyses support the oblique-effect hypothesis such that vection strength during the oblique conditions was weaker than during the cardinal conditions, as evidenced by the lower magnitudes, longer latency, and shorter duration. Next, we directly compared the averages between diagonal oblique data (45° and 135°) and cardinal data (0°, 90°, and 180°) for each vection index. The analyses confirmed that vection strength differed significantly between oblique and cardinal conditions: magnitude: *t*(12) = −3.60, *p* = .004, *r* = .33; latency: *t*(12) = 3.48, *p* = .005, *r* = .84; and duration: *t*(12) = −5.50, *p* = .000, *r* = .59.

Although the results from the statistical analyses support the hypothesis that vection strength has an oblique effect, this conclusion remains somewhat debatable because oblique motion stimuli (30°, 45°, 60°, 120°, 135°, and 150°) were more frequently presented to participants than cardinal motion stimuli (0°, 90°, and 180°). This imbalance in oblique/cardinal condition frequency might itself have biased perception through accumulation effects and led to the observed oblique effect. To address this issue, we repeated the initial experiment but included an equal number of oblique/cardinal conditions (one each: 0° and 30°). Nevertheless, as in the initial experiment, the results from 12 observers (mean age: 33.5 ± 11.1 years; range: 21–54 years; 5 females and 7 males) clearly showed a significant oblique effect: magnitude: *t*(11) = 4.64, *p* = .001, *r* = .76; latency: *t*(11) = 1.80, *p* = .050, *r* = .98; and duration: *t*(11) = 3.62, *p* = .004, *r* = .90. Therefore, we do not think that the imbalanced design was the source of the oblique effect in the initial experiment.

## Experiment 2

The results from Experiment 1 confirmed the effect of motion direction on vection and revealed that this effect varied depending on whether the direction of stimulus motion was cardinal or oblique. However, the effect of motion direction on vection strength could be just reflected the effect of motion direction on motion signal intensity, a finding that has been reported in previous studies (e.g., Dakin et al., 2005; Gros et al., 1998) regardless of the vection-specific process. To respond to this concern, we ran an additional experiment in which participants used magnitude estimation to rate the perceived smoothness of motion and perceived speed instead of vection strength.

### Experimental Method

Stimuli were the same as in Experiment 1, and motion directions were 0° (straight up) and −45° (upper left). Twelve naive observers (mean age: 26.42 ± 5.26 years, range: 21–40 years) participated, and each condition was repeated 4 times for each individual. Participants were instructed to estimate the perceived smoothness and speed of stimuli via visual analogue scales. The values on the analogue scales were translated values between 1 and 100 and averaged across participants.

### Results

**Figure 4.**
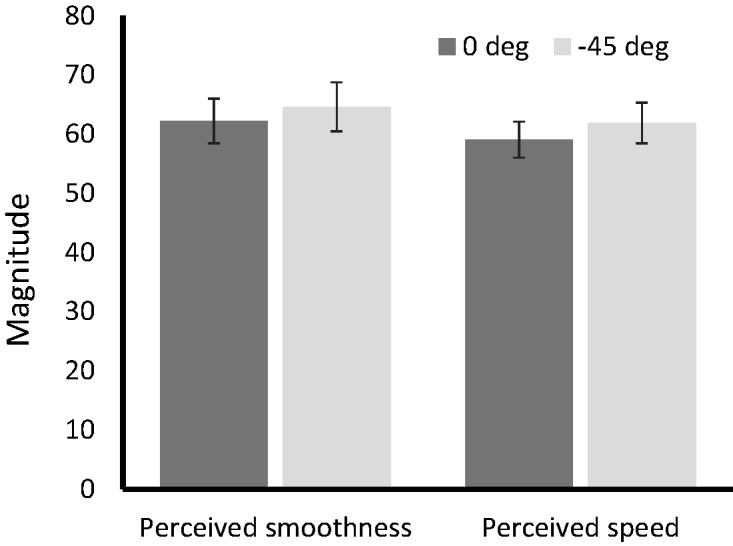
The results for perceived motion smoothness and motion speed (Experiment 2). Error bars indicate standard error (*N* = 12).

The results are shown in Figure 4. Perceptions of these measures did not differ significantly between the 0° and 45° conditions: smoothness: *t*(11) = 0.59, *p* = .57, *r* = .17; speed: *t*(11) = 0.93, *p* = .37, *r* = .27. Thus, oblique effects were not observed for perception of smoothness or speed of motion.

## Discussion

In this study, we examined the effect of motion direction on vection strength. The stimuli were symmetric, and unlike previous studies, the directions of optical flow motion were systematically controlled to cover numerous directions.

The results of Experiment 1 showed a significant effect of motion direction on vection strength. In addition, further statistical analysis and the results from the additional experiment clearly revealed an oblique effect of vection strength which vection strength was weaker in the oblique conditions. Why is vection weaker in oblique directions? One possible explanation is related to the difference in subjective speed. Previous vection studies indicate that the speed of optical flow is a major factor that contributes to vection strength; Tamada and Seno (2015) clearly showed that faster optical flow induced stronger vection. However, it is not clear whether this dependency on speed is related to the actual speed or the subjective speed. Considering the variety of oblique effects, subjective speed could have an oblique effect (or more essential motion gain like motion energy). Furthermore, differences in subjective motion speed could induce an oblique effect of vection strength.

However, the results of Experiment 2 showed that motion signals (subjective smoothness and speed) did not exhibit any oblique effect, despite using the same stimuli as Experiment 1. We can therefore conclude that the oblique effect on vection strength that we observed in Experiment 1 is a specific effect on vection and that the effect cannot be explained by the oblique effects of motion signals that has been shown in previous studies.

The lack of an oblique effect of motion signal in Experiment 2 deserves consideration. One reason for this difference from other studies could be that while our stimuli induced suprathreshold motion signals, those in other studies were near threshold. Our suprathreshold signal likely masked small differences between oblique and cardinal directions.

Hubel and Wiesel (1968) reported orientation sensitive neurons in V1 for a wide variety of directions (both cardinal and oblique). Subsequent studies have reported that more V1 neurons are tuned to horizontal and vertical orientations than to oblique orientations. This was evidenced by an oblique effect in which neurons exhibited higher spatial resolution and contrast sensitivity for lines in the cardinal directions (e.g., De Valois, Yund, & Hepler, 1982; Furmanski & Engel, 2000; Orban & Kennedy, 1981; Orban et al., 1984).

This physiological background should be related to the oblique effect in vection because some studies previously suggested that low-level processing in the visual cortex has an important role in producing vection strength. Gurnsey, Fleet, and Potechin (1998) reported that luminance-defined motion (first-order motion) induced stronger vection than contrast-defined motion (second-order motion). Keshavarz and Berti (2014) reported that neural activity preceded the subjective perception of vection strength (vection onset). They succeeded in recording very early (within 1 second) brain activity related to the subjective experience of vection. Their results also suggest that vection is related to early brain processing in V1. This fact and the less population of cells responding to the oblique orientation in V1 might explain the oblique effect in vection.

Vection studies have indicated that some vection processes are independent from motion perception processes (e.g., Palmisano, Allison, Schira, & Barry, 2015; Seno, Palmisano, Ito, & Sunaga, 2012). For example, Seno et al. (2012) reported that vection direction can be biased by coherent motion stimuli that are not visible and of which the observers are not aware. Studies of vection after effects (VAEs) also indicate the existence of a vection-specific process that might be related to the oblique effect of vection strength. VAEs are after effects similar to motion after effects (MAEs) but are induced by vection perception not motion perception (e.g., Kim & Khuu, 2014; Palmisano, Summersby, Davies, & Kim, 2016; Seno, Palmisano, & Ito, 2011). Although VAEs are closely related to MAEs, the VAE and MAE strength sometimes vary independently from each other, even though identical stimuli are used to induce them. This indicates the existence of a VAE-specific process that is independent from MAEs, implying the existence of a vection-specific process that is different from motion processes.

Our suprathreshold optical flow induced an oblique effect on vection strength but did not induce effects on other motion signals (as shown by Experiment 2). This indicates that the optical flow was processed almost symmetrically between oblique and cardinal directions in terms of motion but asymmetrically in terms of vection. Therefore, long exposure to the flow might induce symmetrical inhibition in motion processing but asymmetrical inhibition in vection processing. This difference can be observed behaviorally as symmetry in MAEs and asymmetry in VAEs and is a very interesting topic for future study.

When looking at the results for the cardinal directions, we did not find any differences in vection strength between them. This contrasts with previous studies, which have reported that vertical motion induced stronger vection than horizontal motion (Ito, 2004; Ito & Fujimoto, 2003; Telford & Frost, 1993; Trutoiu & Mohler, 2009) and that upward motion induced stronger (or weaker) vection than downward motion (stronger: Seya et al., 2015 and Telford & Frost, 1993; weaker: Kano, 1991). However, this does not necessarily indicate a conflict. Although we did not find significant differences, the trend was for vertical conditions (0° and 180°) to be systematically stronger than the horizontal condition (90°) and for the up condition (0°) to be stronger than the down condition (180°) for all three indices of vection strength. In addition, the results suggest that the difference in vection strength between the cardinal directions and oblique directions is relatively larger than the difference among cardinal directions. Finding little or no difference in the effects of cardinal directions on vection strength might reflect the suggestion in Keshavarz et al. (2017) that differences in motion direction between horizontal and vertical directions is not a crucial factor in vection perception. In contrast, the larger differences between cardinal and oblique directions can reflect a crucial factor. The studies of asymmetry in vection strength for cardinal directions also used suprathreshold motion stimuli, and all parameters except direction were identical to our experiment. The similarity between our experiment and the experiments described earlier might indicate that as with the oblique effect on vection strength, asymmetry in cardinal directions might also be induced by vection-specific processes, not motion processes.

## Conclusion

We found that vection strength depends on the direction of optical flow motion; optical flow in oblique directions induced weaker vection than vertical or horizontal flow. This oblique effect could not be explained by any differences in the motion signal, which itself did not show any oblique effect. Thus, vection strength depends on direction, while motion signals do not—a distinction reflected in the different types of processing needed for motion perception and vection. Although this kind of dual measurement with the same stimuli has not been common in previous vection studies, we think that in the future, this simple method has the potential to reveal additional properties of the vection process.

## Note

1 The tendencies found in the results did not change depending on whether the two authors were included or excluded from the analysis. We observed the same significant main effects with almost the same *F* and *p* values.
